# Supplementary material for: Gene flow and species delimitation in fishes of Western North America: Flannelmouth (Catostomus latipinnis) and Bluehead sucker (C. Pantosteus discobolus)
Source: Ecol Evol. 2020 Jun 17;10(13):6477–93. doi: 10.1002/ece3.6384 (PMC7381754; doi:10.1002/ece3.6384)
Supplement: Supplementary file 1 — Figure S1‐S2 [file ECE3-10-6477-s001.pdf]

A

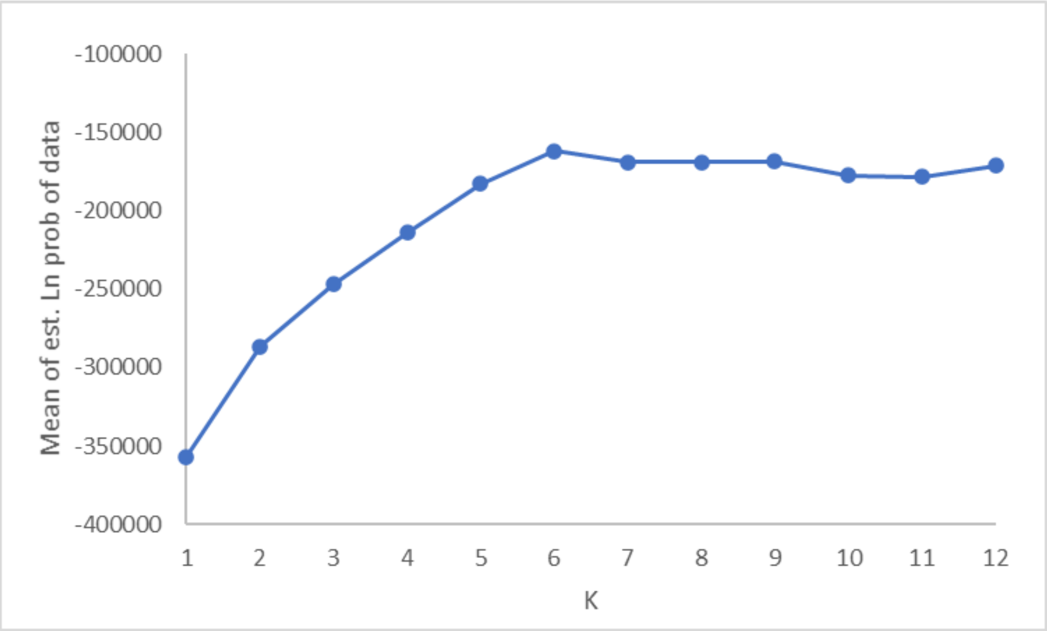

B

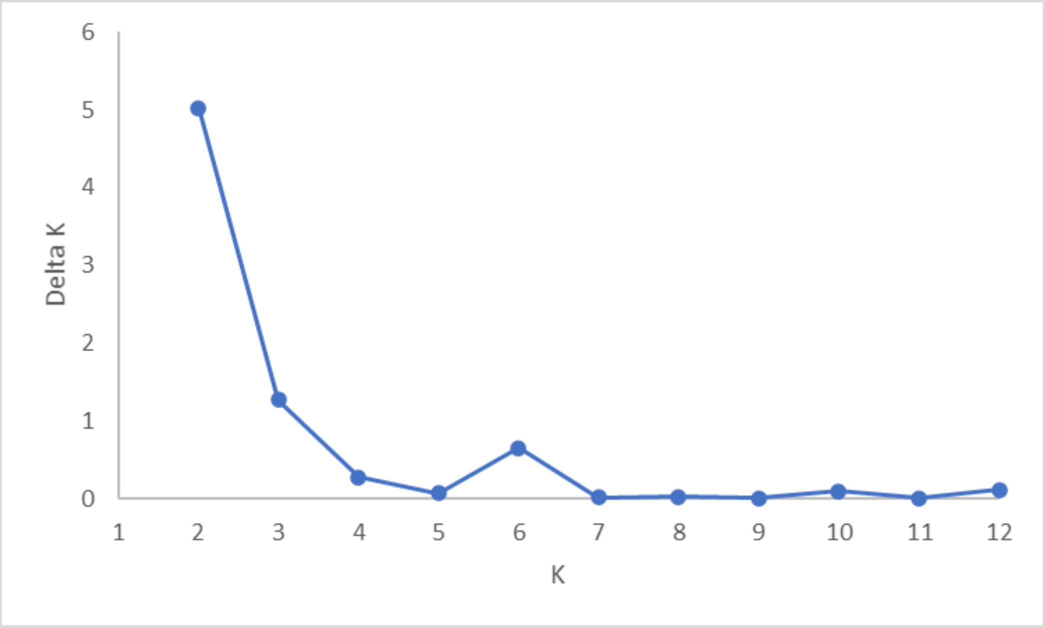

Supplemental Figure 1: (A) Mean natural log probabilities of data across 15 replicates against number of clusters (K) for the *Pantosteus* subgenus Structure runs. (B) Delta K (as calculated per Evanno et al. 2005) across 15 replicates against number of clusters (K) for the *Pantosteus* subgenus Structure runs.

A

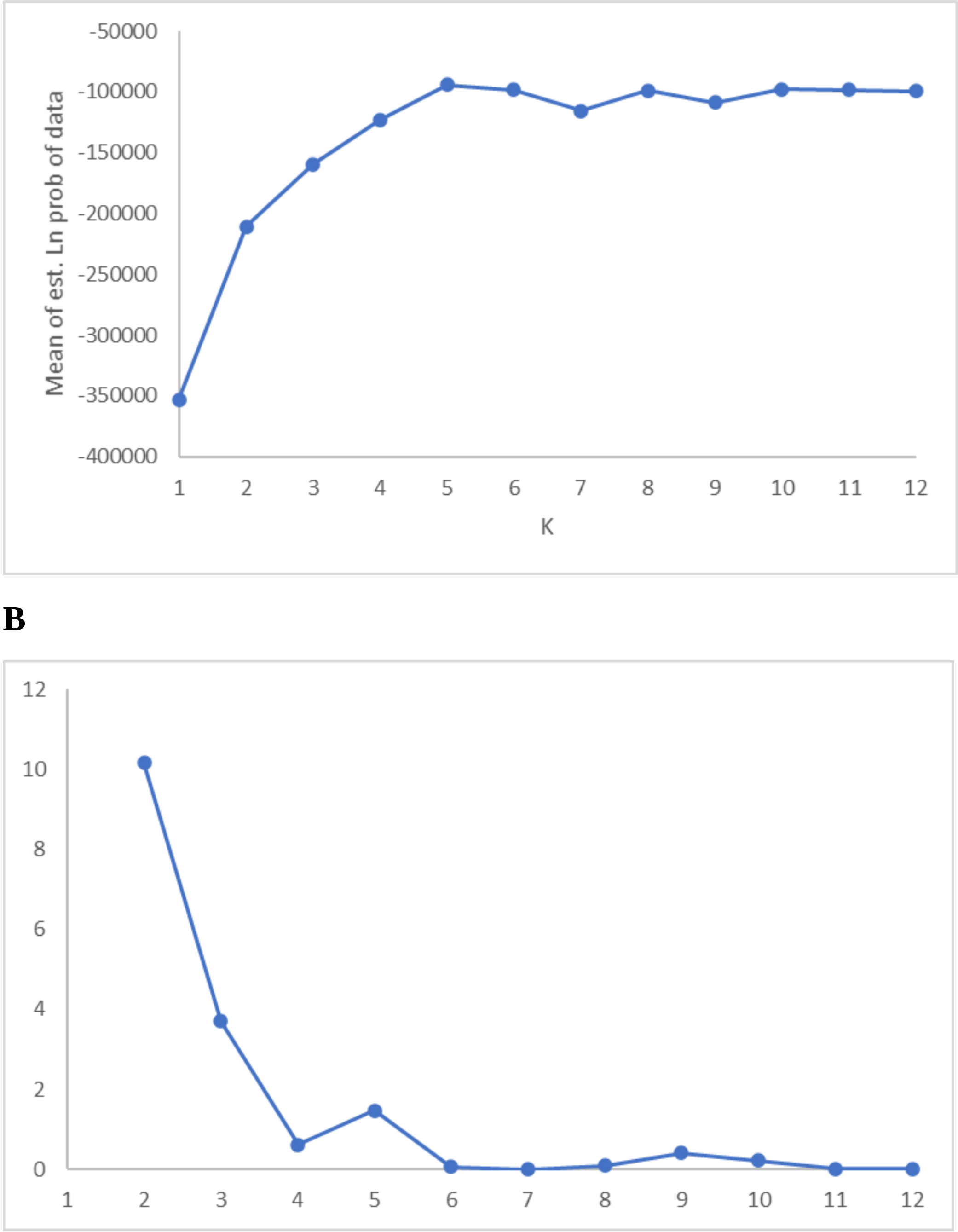

Supplemental Figure 2: (A) Mean natural log probabilities of data across 15 replicates against number of clusters (K) for the *Catostomus* subgenus Structure runs. (B) Delta K (as calculated per Evanno et al. 2005) across 15 replicates against number of clusters (K) for the *Catostomus* subgenus Structure runs.
